# A Study of Physical Activity, Frailty, and Health-Related Quality of Life Among Community-Dwelling Older Adults in Taiwan

**DOI:** 10.1097/JNR.0000000000000402

**Published:** 2020-09-16

**Authors:** Pei-Shan LI, Chia-Jung HSIEH, Nae-Fang MIAO

**Affiliations:** 1 MSN, RN, Department of Nursing, Taipei Veterans General Hospital Respiratory Intensive Care Unit, and Doctoral Student, College of Nursing, National Taipei University of Nursing and Health Sciences, Taipei, Taiwan, ROC; 2 PhD, RN, Associate Professor, School of Nursing, College of Nursing, National Taipei University of Nursing and Health Sciences, Taipei, Taiwan, ROC; 3 PhD, RN, Associate Professor, Post-Baccalaureate Program in Nursing, College of Nursing, Taipei Medical University, Taipei, Taiwan, ROC.

**Keywords:** community-dwelling older adults, physical activity, frailty status, health-related quality of life

## Abstract

**Background:**

Health-related quality of life (HRQoL) in community-dwelling older adults increases as physical activity improves, and age-related frailty has a negative effect on HRQoL. Research on these associations is lacking.

**Purpose:**

The aims of this study were to (a) analyze the effect of demographic characteristics on HRQoL, (b) explore the correlation between physical activity and HRQoL, (c) analyze the effect of frailty on HRQoL, and (d) investigate the potential predictors of HRQoL in community-dwelling older adults.

**Methods:**

In this cross-sectional study, a convenience sample of 150 older adults was recruited from community care sites in Shilin and Beitou Districts in Taipei City, Taiwan. Data were collected at baseline using a demographic characteristics datasheet, the Center for Epidemiologic Studies Depression Scale, the Physical Activity Scale for the Elderly, and the 12-Item Short Form Health Survey. The Senior Fitness Test and hand-grip strength test were also performed. Student *t* test, chi-square test, analysis of variance, Pearson correlation coefficient, and hierarchical regression were applied to analyze the statistical results using IBM SPSS Statistics Version 22.0.

**Results:**

Being of older age, experiencing a higher number of falls, having more chronic diseases, and having a higher body mass index were identified as factors that significantly affect HRQoL. Moreover, HRQoL was found to be significantly affected by the performance of physical activity or status of frailty. Furthermore, the prefrail period was shown to be an important predictor of HRQoL after adjusting for demographic variables, history of chronic illness, history of falls, and physical activity.

**Conclusions/Implications for Practice:**

In this study, HRQoL was found to be significantly affected by upper limb dysfunction and the prefrail period. Community health promotion activities should focus greater attention on the physical functioning of older adults. Furthermore, providing information on age-related frailty and promoting active participation in community activities may increase the attention given by community-dwelling older adults to physical fitness and quality of life.

## Introduction

Population aging is a worldwide phenomenon in the 21st century. Taiwan is currently experiencing a rate of population aging that is significantly higher than other Asian countries, with about 16% of the population now classified as older adults (≥ 65 years old; Department of Household Registration, Ministry of the Interior, Taiwan, ROC, 2020). The onset of early old age is marked by individuals beginning to lose, ultimately, between 30% and 40% of their muscle mass (Haber, 2013). Weakening in skeletal muscle strength or muscle atrophy induces fatigue, especially in the back and/or lower limb muscles. Decreased activity, inability to move quickly, and dysfunction in balance or walking increase the risks of accidental falls and related complications. Different variances and effects may be observed based on age and gender as well as health status, chronic disease that resulted in physical activity habits, muscle mass, cardiopulmonary fitness, and flexibility (Milanović et al., 2013). The function of upper limb muscles is dependent on the execution of free movements and fine movements. However, the functioning of this muscle group is rarely a focus of attention in the literature. On the whole, aging leads to reduced physical activity, emotional disorders, social isolation, and increased risk of chronic diseases and ultimately results in the gradual decline in function of multiple organs and physiological systems. The presence of geriatric syndromes that are concomitant with cognitive disorders has also been correlated with disability, hospitalization, and increased risk of death (Davis, Bryan, Li, et al., 2015). Higher frequency of chronic diseases increases the complexity of healthcare and thus increases emotional and physical ailments as well as financial burdens and stress (Parker et al., 2014). All of these factors affect the quality of life experienced by older adults.

Engaging in regular, physical activity is an important factor affecting the quality of life of older adults. Previous studies have highlighted a strong association between physical functioning and health-related quality of life (HRQoL) in older adults, which indicates that physical functioning may help predict future hospitalization and care needs (Sartor-Glittenberg et al., 2014; Shiu et al., 2013; Van Dyck et al., 2015; Wang & Li, 2011). Several studies have shown that increasing the physical activity of older adults may lower the risk of falling and physical disabilities and slow down the aging process (Bauman et al., 2016). Encouraging regular physical activity among community residents is regarded as an important, nonpharmacological treatment. Especially in older adults, increasing the amount of physical activities performed during leisure time helps maintain health and prevent declines in quality of life, highlighting the important link between physical activity and HRQoL (Halaweh et al., 2015). Similar to muscular strength, muscular endurance and dynamic homeostasis have been identified as risk factors of frailty in studies on older community residents (Chang et al., 2014; Sewo Sampaio et al., 2016). Thus, further investigation of the impact of physical activity on quality of life in older adults is necessary.

Frailty leading to a decline in physical function is one of the manifestations of geriatric syndrome. As age increases, people who become frail will experience further deterioration in organ function, inducing negative effects across various bodily systems, the outcomes of which will affect health and quality of life (Rizzoli et al., 2013). Frailty leads not only to many complicated problems but also to a decline in independent living skills, resulting in the inability to prepare meals or to undertake activities. Insufficient protein intake reduces muscle mass, whereas insufficient caloric intake for daily needs leads to malnutrition. These conditions further exacerbate weight loss and cognitive impairment, increasing the need for assistance for daily life activities such as dressing, eating, toileting, and moving. The outcomes of frailty include falls, injuries, hospitalization, and comorbidities. Moreover, frailty is a predictor of poor prognoses in terms of disability, hospitalization, residential institutions, and death (Landi et al., 2010). As accidents endanger quality of life in older adults, the importance of healthcare increases. Relevant studies have shown that varying degrees of frailty may affect HRQoL (Chang et al., 2012) and that frailty status is strongly associated with older community residents and their HRQoL (Amer et al., 2015).

Physical fitness reflects the health status of an individual. Poor health and frailty lead to declining physical function. Pain, disability, depression, chronic disease, and frailty status in older adults have all been identified as significant indicators of quality of life (Davis, Bryan, Best, et al., 2015). Thus, it is vital to investigate the factors that influence physical activity, frailty status, and HRQoL in older adults. Health assessments have been designed to assess the impact of certain factors on quality of life. These assessments address dynamic, subjective, and multifaceted items; physiological, psychological, emotional, and social functions; and psychological emotions, cognitive functions, economic status, and mental state (Bakas et al., 2012). The health performance may intimate the functional health status of subjects via their subjective feelings (Davis, Bryan, Li, et al., 2015). For older adults, living a healthy, high-quality life is much more important than simply increasing lifespan. Thus, it is essential to achieve a successful and satisfying life in later years.

As mentioned above, strong correlations among physical activity, frailty status, and HRQoL in older, community-dwelling adults have been documented in previous studies (Amer et al., 2015; Rizzoli et al., 2013; Sartor-Glittenberg et al., 2014; Wang & Li, 2011). However, the information in these studies regarding HRQoL, specifically in relation to physical activity and frailty, is insufficient for older adults. To fill this gap, the participants recruited in this study were older adults living in community settings. The aim of this study was to understand the physical activity, frailty status, and HRQoL of the participants; to analyze the associations among these three aspects; and to investigate the important prognostic factors of HRQoL. The hypotheses of this research were as follows: (a) Different demographic characteristics lead to significant differences in HRQoL; (b) physical activity and HRQoL are positively correlated; (c) significant differences in frailty status will be found with respect to HRQoL; and (d) demographic characteristics, physical activity, and frailty status are significant predictors of HRQoL. Therefore, the purposes of this research article were to (a) analyze the differences in participants' demographic characteristics and HRQoL, (b) explore the correlation between physical activity and HRQoL, (c) analyze the differences in frailty status and HRQoL among the participants, and (d) investigate the important factors that may be used to predict HRQoL in this population. The results may help healthcare professionals use the physical activity and frailty status of older adult patients to assess the impact of these factors on their patients' HRQoL and recommend appropriate caring activities to improve HRQoL.

## Methods

### Study Design

This was a cross-sectional study. Convenience sampling was used to collect data from older adults living in the community who were at least 65 years old and who were involved in community-care and care-related activities in the Shilin and Beitou Districts of Taipei City, Taiwan. The inclusion criteria were as follows: (a) aged ≥ 65 years; (b) currently living in Shilin or Beitou District in Taipei City; (c) able to communicate in Mandarin or Hokkien Chinese; (d) conscious, agreeable, and willing to join in all of the research activities and to complete the questionnaire evaluation; and (e) able to walk either independently or with a walker. The exclusion criteria were as follows: (a) unable to move independently (e.g., those in long-term bed rest, who regularly used a wheelchair, or were unable to walk alone); (b) hearing, visual, or cognitive impairments that prevented regular cooperation with data collection activities; (c) long-term residency at a caring institution; and (d) participation in a cross-sectional or interventional study of physical activity, function, or fitness within the previous 6 months.

### Participants

The minimum number of participants in this study was set according to the program setting of G*Power 3.1.9.2 (Heinrich Heine Universitat, Dusseldorf, Germany). The *f*^2^ effect size, alpha value, power value, number of independent variables, and estimated effective number of participants were 0.15, 0.05, 0.8, 13, and ≥ 131, respectively. After estimating a potential attrition rate of 15%, the actual number of participants enrolled was 150. Fifteen participants took part in the pilot studies. The study was conducted between November 6, 2017, and May 31, 2018. All of the participants provided their full name and date of birth. After explaining the research workflow and purpose, the participants were asked to fill in the structured questionnaire during a face-to-face interview session. In addition, a community caring district-based arrangement acquisition was conducted to evaluate each participant's level of physical fitness.

### Measures

#### Dependent variables

Ten questions in the structured questionnaire gathered demographic information, including age, gender, marital status, educational level, living conditions, source of income, tobacco and alcohol use, chronic illness history, fall history, and body mass index (BMI).

The structured questionnaire on physical activity included the Physical Activity Scale for the Elderly (PASE) and the Senior Fitness Test (SFT). In the PASE, the paired item of the frequency and duration of the physical activity practiced per week on each of the 12 activities was multiplied and then divided by 7 to obtain the average daily activity level. Next, sums of the number of physical activities performed by different items were measured by multiplying the above value by a constant. The PASE was graded on a scale of 0–360, with higher scores representing greater physical activity. Information regarding physical activity and “insufficient quantity of physical activity” in the tests of the frailty phenotype were collected. Both the reliability (*r* = .892, *p* < .05) and validity (*r* = .379, *p* < .05) of the Chinese version of the PASE have been shown (Ku et al., 2013). The SFT, used to measure body function, includes a 30-second chair stand, arm curl, a 2-minute step test, chair sit-and-reach, back scratch, a single-leg standing test, and an 8-foot seated up-and-go test (Jones &Rikli, 2002). Muscle strength, muscle endurance, flexibility, agility, and dynamic balance were adopted as indicators in this study. The upper limb flexibility test was used to measure the distance in inches between fingertips. When the fingertips were unable to touch in the back, the value of the distance was negative. In cases where a participant's two-finger fingertips cannot overlap, the greater the width of the gap (the negative value increases), the worse the upper limb flexibility. Dynamic balance ability refers to the ability to maintain physical balance through limb adjustments during exercise. More time spent in dynamic balance is associated with worse functional mobility. Impaired dynamic balance reduces the ability to balance during exercise (Jones & Rikli, 2002).

The SFT may be used to measure strength, flexibility, and balance in the context of the daily living activities of older adults. The measurement standards used in this study were adopted from the National (Taiwan) Survey of Older Adults, and comparisons are made in the text between the data from this study and the functional fitness data published for this survey (Ministry of Education Sports Department, Taiwan, ROC, 2015).

Frailty status is judged based on the frailty phenotype of the Cardiovascular Health Study (CHS). Older adults with no frailty phenotype are classified as “not frail,” those with one to two phenotypes are classified as “prefrail,” and those with three or more phenotypes are classified as “frail.” The frailty criteria are as follows: (a) weight loss, that is, BMI lower than 18.5 kg/m^2^ (“lightweight” as defined by the National Health Council) or unintentional reduction in 5% body weight or 3 kg over a 1-year period; and (b) poor grip strength, measured by handgrip dynamometer while the participant was in a standing position, using the dominant hand twice, with the higher (best) value of the two recorded. The results were classified based on the gender and BMI criteria used in the CHS and in reference to local standards (different standards for men and women; Lin et al., 2011). (c) Feeling unable (physically) to do anything (as noted in the structured questionnaires of the epidemiological study) was assessed. Two questions, based on the Center for Epidemiologic Studies Depression Scale, were designed to assess depression (“*I felt that everything I do is an effort*” and “*I cannot get going*”). If a participant reported agreement with either statement for a period lasting 3 days or more during a 1-week period, the participant was assessed as “depressed.” The reliability of the Chinese version of the Center for Epidemiologic Studies Depression Scale has been reported as .78 or higher (Yu et al., 2011). (d) Slow walking speed was assessed for participants whose walking speed was in the slowest 20% of the sample in the physical function test according to gender and height criteria used in the CHS and referencing current government standards (Lin et al., 2011). (e) Insufficient physical activity was assessed using the physical activity scores of participants. Those in the lowest 20%, according to gender criteria used in the CHS and referencing current government standards, were identified as being insufficiently physically active (Lin et al., 2011).

#### Independent variables

A Short Form-12 (SF-12) structured health questionnaire was used to estimate the HRQoL of the participants. These outcomes were calculated using 12 self-evaluated short questions. Scores for each were converted to a 0–100 scale. Two facet scores and the overall total scores of the physical component scale and the mental component scale were adopted as the basic indicators of HRQoL. A score of 0 represented the worst HRQoL, and a score of 100 represented the best HRQoL. The Cronbach's α value for the Chinese version was reported as greater than .8, whereas the Cronbach's α values of physical and physiological health were .80 and .72, respectively (Liu & Chiou, 2012).

#### Ethical considerations

This study was approved by the joint institutional review board (JIRB No. 17-S-016-1). In compliance with the principle of acceptance, all of the study subjects were fully informed regarding the nature of the research and were informed of their right to withdraw without prejudice at any time. The original data gathered were maintained for a certain period to facilitate checking and additional objective and detailed analyses.

#### Statistical analysis

Statistical analysis was performed using IBM SPSS Statistics for Windows Version 22.0 (IBM, Inc., Armonk, NY, USA) Descriptive statistics were used to calculate the maximum, minimum, percentage, mean, and standard deviation. Inferential statistics, including independent sample *t* test, chi-square test, analysis of variance, Pearson correlation coefficients, and hierarchical regression, were used for statistical analyses.

## Results

Two thirds (32.00%, *n* = 48) of the participants experienced one chronic disease, and 68.67% reported experiencing no falls during the past 1-year period. Increases in the number of chronic diseases and number of falls were both associated with lower HRQoL scores (*F* = 5.20, *p* < .01, and *F* = 3.93, *p* < .01, respectively; Table 1). The average age of the participants was 76.09 ± 6.53 years, the average BMI was 24.63 ± 3.60 (Table 2), and the average HRQoL score was 73.24 ± 17.76. Older age and higher BMI were both associated with lower HRQoL (*r* = −.24, *p* < .01, and *r* = −.19, *p* < .05, respectively). Better lower limb flexibility and upper limb flexibility were found to correlate positively with HRQoL (*r* = .25, *p* < .01, and *r* = .37, *p* < .01, respectively). Dynamic equilibrium was found to correlate negatively with HRQoL (*r* = −.40, *p* < .01), indicating that participants who spent more time in dynamic equilibrium had poorer HRQoL (Table 2).

**Table 1:** Demographic Characteristics of Participants, According to HRQoL (*N* = 150)

Demographic Characteristic	Mean	*SD*	*F/t*	Multiple Post Hoc Comparison
Gender			−0.29	
Male	72.61	21.46		
Female	73.53	15.83		
Marital status			0.26	
Married	74.14	17.03		
Divorced or widowed	71.07	19.44		
Educational level			0.81	
Illiterate	64.93	26.89		
Elementary or below	71.91	18.58		
Junior and senior high school	73.92	17.99		
College or above	75.91	13.70		
Living conditions			0.87	
Living alone	74.42	20.77		
Living with spouse	74.22	17.21		
Living with children and relatives	69.63	17.73		
Financial status			0.58	
Stable income with allowance	78.22	15.26		
With financial support by juniors	70.53	18.11		
Pension or savings	73.59	17.89		
Tobacco and alcohol use			0.71	
No use at all	73.72	17.54		
Used tobacco or wine	70.19	18.95		
Chronic illness history			5.20**	
① None	81.99	15.60		① > ③ > ④
② One	74.31	16.29		② > ③ > ④
③ Two	73.26	13.35		
④ Three or more	65.41	21.54		
Fall history (falls in 1 year)			3.93**	
① None	76.15	15.32		① > ②
② 1–2 times	68.87	19.63		① > ③
③ 3–4 times	57.64	18.05		
④ 5 or more times	63.33	33.22		

***Note.*** HRQoL = health-related quality of life.

**p < .01.

**Table 2:** Correlation Analysis of Age, BMI, Physical Activity, and HRQoL (*N* = 150)

Physical Activity	Mean	*SD*	Male/Female PR	*r*
Age	76.09	6.53		−.24**
BMI	24.63	3.60		−.19*
Physical activity				
Lower limb muscle strength/muscle endurance	16.55	6.05	70 / 80	.33**
Upper limb muscle strength/muscle endurance	18.39	5.70	50 / 55	.41**
Static balance	15.20	10.70	60 / 75	.27**
Cardiorespiratory endurance	93.02	27.45	65 / 70	.23**
Lower limb flexibility	4.53	11.59	70 / 55	.25**
Upper limb flexibility	−7.11	11.09	65 / 40	.37**
Dynamic balance and agility	6.92	2.59	60 / 70	−.40**
Physical activity	95.70	48.77		.40**
HRQoL	73.24	17.76		1.00

***Note.*** BMI = body mass index; HRQoL = health-related quality of life; PR = percentile rank.

*p < .05. **p < .01.

A significant correlation was found between HRQoL and physical activity. Compared with results of the physical fitness of the older adults, low percentage was obtained in the percentile norm of the arm curl (upper limb muscle/muscle endurance) and the scratch-back test (upper limb flexibility). The better performance obtained in the upper limb muscle strength/muscle endurance and the upper limb flexibility, the higher the level of HRQoL of the participants (*r* = .41, *p* < .01; *r* = .37, *p* < .01; Table 2).

Most (44%, *n* = 66) of the participants in this study were classified as “not frail.” The prevalence of frailty in this community was found to be 18.00%. The statistical differences in HRQoL based on the clinical stage of frailty (*F* = 20.44, *p* < .01) are highlighted in Table 3. A hierarchical regression analysis, adjusted for age and BMI, was performed to investigate the prognostic factors of the HRQoL. In Model 2, having two chronic illness histories, having three or more chronic illness histories (β = −0.21, *p* < .05, and β = −0.34, *p* < .01, respectively), and having a history of one to four falls were each associated with a lower HRQoL score (β = −0.15, *p* < .05, and β = −0.17, *p* < .05, respectively).

**Table 3:** Analysis of Variances for Frailty Status and HRQoL (*N* = 150)

The Frailty Status	Mean	*SD*	*F*	Multiple Post Hoc Comparison
The clinical stage of frailty			20.44**	① > ② > ③
① No frailty	81.69	12.42		
② Prefrail	69.77	17.46		
③ Frailty	59.88	19.28		

***Note.*** HRQoL = health-related quality of life.

**p < .01.

**Figure 1:**
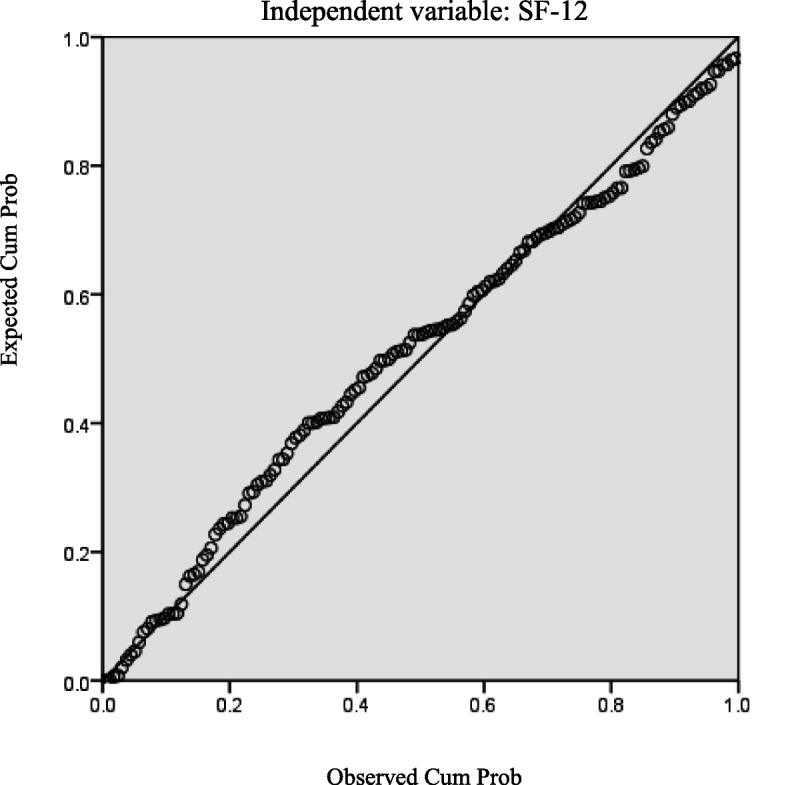
Normal P–P Plot of Health-Related Quality of Life (Multiple Hierarchical Regression Analysis)

In Model 3, after adjusting for chronic illness and fall histories, three variables were associated with higher HRQoL scores: upper limb strength/muscle endurance, upper limb flexibility, and amount of physical activity (β = 0.24, *p* < .05; β = 0.20, *p* < .05; and β = 0.23, *p* < .01, respectively). In Model 4, after adjusting for demographic characteristics, history of chronic illness, history of falls, and physical activity, the analysis revealed that the prefrail stage was associated with a lower HRQoL score (β = −0.22, *p* < .05). The total variation explained by the full statistical regression models was 31.70% (adjusted *R*^2^ = .32), and the final results are shown in Table 4. The full model showed statistically significant results according to the Shapiro–Wilk normality test (*R*^2^ = .40, *p* > .05), and the results showed normality in multiple regression (Figure 1).

**Table 4:** Multiple Hierarchical Regression Analysis of Demographic Characteristics, Physical Activity, Frailty Status, and HRQoL (*N* = 150)

Variable Item	HRQoL								Collinearity Statistics (VIF)
	Model 1		Model 2		Model 3		Model 4		
	β	*t*	β	*t*	β	*t*	β	*t*	
(Constant)		7.61		7.16		3.27		3.40	
Demographic characteristics									
Age	−0.21	−2.65**	−0.16	−2.02*	−0.01	−0.12	0.03	0.27	1.78
BMI	−0.15	−1.90	−0.10	−1.20	−0.01	0.12	−0.03	−0.40	1.41
Chronic illness history (ref: no history)									
One			−0.20	−1.88	−0.15	−1.51	−0.11	−1.11	2.01
Two			−0.21	−2.07*	−0.13	−1.32	−0.07	−0.72	2.01
Three or more			−0.34	−3.29**	−0.21	−2.09*	−0.15	−1.48	2.29
Fall history (ref: no history)									
1–2 times			−0.15	−1.99*	−0.17	−2.41*	−0.19	−2.63*	1.12
3–4 times			−0.17	−2.13*	−0.10	−1.34	−0.10	−1.35	1.25
5 or more times			−0.04	−0.52	0.04	0.46	0.04	0.57	1.33
Physical activity									
Lower limb muscle strength/muscle endurance					−0.03	0.30	−0.01	−0.05	2.40
Upper limb muscle strength/muscle endurance					0.24	2.54*	0.22	2.32*	1.96
Static balance					−0.12	−1.13	−0.15	−1.44	2.30
Cardiorespiratory endurance					0.02	0.19	−0.02	−0.27	1.47
Lower limb flexibility					−0.01	0.16	−0.03	−0.04	1.47
Upper limb flexibility					0.20	2.04*	0.18	1.88	2.01
Dynamic balance and agility					−0.14	−1.28	−0.12	−1.02	3.18
Physical activity					0.23	2.68**	0.18	2.03*	1.66
Frailty status (ref: no frailty)									
Prefrail							−0.22	−2.59*	1.54
Frailty							−0.18	−1.58	2.83
*R*		.28		.44		.61		.63	
*R*^2^		.08		.19		.37		.40	
*R*^2^ change		.07		.15		.29		.32	
Adjusted *R*^2^		.08		.11		.18		.03	
*F*		6.33**		3.24**		4.65***		3.42*	

***Note.*** BMI = body mass index; HRQoL = health-related quality of life; ref = reference; VIF = variance inflation faction.

*p < .05. **p < .01. ***p < .001.

## Discussion

This is the first study designed to examine the correlation among the three aspects of physical activity, frailty status, and HRQoL in older community-dwelling adults. The results, verified by statistical analyses, confirm that demographic characteristics significantly affect HRQoL in this population. The statistical analysis performed on the data in this study showed that age, BMI, history of chronic diseases, and history of falls each significantly affect HRQoL.

The participants in this study had a significantly older mean age than the respective participants in two related studies (Mulasso et al., 2014; Wang & Li, 2011). Similar to related studies conducted in Taiwan, in this study, age was found to have a profound impact, whereas history of chronic disease was found to have only a minor effect on HRQoL (Li, 2005). In addition, in a study on older community-dwelling women in Brazil, a history of falls was identified as a significant predictor of HRQoL (Sewo Sampaio et al., 2016). Moreover, Purath and colleagues mentioned that high BMI has a significant impact on HRQoL (Purath et al., 2009).

On the basis of the findings in this study, age and falls affect HRQoL at differing degrees, independent of the districts in which the participants lived. Chronic diseases and BMI have an uncertain but significant effect on HRQoL. In older adults, HRQoL is determined by physical health, mental health, mental adjustment, movement capability, social connections, personal beliefs, and other factors (Halaweh et al., 2015). Furthermore, various demands and targets set in daily life indirectly affect the results. The results of this study revealed that age, BMI, history of falls, and history of chronic diseases may be used as indicators for evaluating HRQoL in older adults.

In addition, the results of this study showed worse HRQoL scores for participants with poor lower and upper limb muscle strength/muscle endurance, poor limb flexibility and flexibility, and poor dynamic balance and agility. These results are similar to those of another study on exercise in older adults that focused on limb flexibility (Justine et al., 2011), the findings of which supported poor flexibility as an indicator of muscle and joint stiffness, which restricted overall physical activity and potentially affected quality of life. In other words, limb tightness hinders physical activity, which compounds problems and leads to a continual decrease in flexibility (Justine et al., 2011). This indicates that reduced limb flexibility, which is common in natural aging, not only contributes to further declines in limb flexibility but also greatly affects all aspects of physical activity and, consequently, HRQoL.

Impaired dynamic balance ability inhibits an individual to balance during exercise (Jones & Rikli, 2002). In this study, the participants who performed better in physical activities had a better HRQoL. Similar to the results obtained in another cross-sectional study (Kim & Lee, 2018), HRQoL in this study was affected by age and levels of physical activity. Likewise, “older adults with better physical activity performance have better HRQoL” was found in another intervention study (Chan et al., 2016). Similar to the findings of a literature review study (Purath et al., 2009), declining physical fitness in terms of muscle endurance, balance, softness, cardiovascular endurance, and physical activity were all associated with decreased HRQoL. Finally, variations in the rate of aging may cause different outcomes in the evaluations of physical activity. In short, physical activity was shown to have a direct impact on HRQoL.

This study also showed that participants who were not yet frail had a significant and negative correlation with HRQoL. Possible explanations for this finding include the low number of frail subjects. Individuals in the early stages of frailty and those who are classified as frail are expected to have different levels of HRQoL (Mulasso et al., 2014). Another explanation is that aging involves limited functional activity, including the geriatric status of frailty. Prefrail status was closely linked to HRQoL in this study. Especially in the early stages of frailty, the participants were usually unstable and facing difficulties in adaptation, which has a strong, indirect impact on life quality (Amer et al., 2015).

Similar to other frailty studies conducted on older adults in Taiwan, in this study, more severe frailty was directly associated with a stronger impact on HRQoL (Chang et al., 2012). On the basis of the clear relationship established between frailty and HRQoL in this study, greater attention should be paid to the ongoing changes in frailty status when caring for older community-dwelling adults. On the basis of the results of another interventional research study, we know that exercise and nutrition may improve frailty, bone density, and Vitamin D serum concentration in older community-dwelling adults. Improvements in the physical activity of older adults who are in different stages of frailty have been noted in previous studies, especially when guidance and intervention were provided by case managers (Chen, 2015). Before and after implementing interventions in older community-dwelling adults affected by frailty, it is important to evaluate frailty status and physical function to track changes in frailty and the efficacy of interventions in improving HRQoL.

Another objective of the study was to identify the prognostic factors affecting HRQoL in the target population. Using multiple regression analysis, age, history of chronic disease, and history of falls were identified as the significant prognostic factors of HRQoL. Earlier studies similarly found that age, falls, and medical conditions are significant prognostic factors (Mulasso et al., 2014; Sewo Sampaio et al., 2016). As age and chronic diseases affect HRQoL, it is important to pay greater attention to treatment and interventions in older adults. To our knowledge, falls are related to more than simply lower-limb strength. Studies have shown that history of falls may predict HRQoL (Sewo Sampaio et al., 2016), which is a finding similar to that of this study. Moreover, studies from countries other than Taiwan have reported similar findings, confirming that physical activity may affect HRQoL in older adults (Sartor-Glittenberg et al., 2014; Sewo Sampaio et al., 2016; Wanderley et al., 2011).

In contrast to the findings of previous studies (Wang & Li, 2011), upper limb strength, muscular endurance, and frailty status were identified in this study as the most significant prognostic factors. This finding highlights the importance of hand-grip strength to older adults. When providing exercise training to older adults, in addition to quadriceps muscle strength, appropriate stretching exercises should be provided to improve muscle tightness, reduce muscle discomfort, and resolve bad posture to promote physical balance.

Physiological analysis has found that messages regarding human physical activity are conveyed to the cerebral cortex through sensory receptors in the body. The cerebral cortex contains the primary motor cortex of the skeletal muscle, controlling a broader spectrum of the movement of the upper limbs than the related area of the lower limbs. The studies monitoring motor system regions as subjects played a piano revealed that the primary motor area of the premotor cortex and cerebellum significantly increased brain plasticity (Tavor et al., 2019). This suggests that upper limb activity increases the primary motor area of cerebral cortex as well as blood flow to the primary somatosensory cortex. This supports the theory that a decline in upper limb function may cause part of the cortex to atrophy. The function of the upper limbs is crucial to movement, balance, and socialization. The difference between upper and lower limb function implies different degrees of influence on HRQoL in older adults. When engaging in physical activity, the lower limbs are primarily responsible for supporting body weight. In contrast, the upper limbs not only need to perform many physical activities and facilitate free movements but also must perform technical fine-motion tasks. Among older community-dwelling adults who have functional motor defects in their lower extremities, many support their bodies only by relying on their upper limbs aided by a walker or wheelchair. Functional motor defects affecting the upper limbs prevent individuals from engaging in normal activities of daily life such as changing clothes, eating, and cleaning. In this situation, they must rely entirely on others to help them perform daily activities. In turn, they will encounter various types of disequilibrium in their lives. As a result, the burden and pressure on the individual will increase. This is in line with the conclusion that hand injuries usually cause negative psychological effects (MacDermid et al., 2018). The results of this study support the importance of upper limb functions.

Upper limb muscle strength/endurance, upper limb flexibility, and prefrail status were identified in this study as significant prognostic factors of HRQoL, echoing the results of similar findings that grip strength significantly affects HRQoL in older adults (Wanderley et al., 2011) and confirming that reductions in upper limb muscle strength are associated with poorer HRQoL. Most of the participants in this study were retired civil servants or teachers. Their acclimation to doing paperwork, which requires upper limb dexterity, may lead to their having more intense, negative feelings about declines in upper limb function. Other studies have shown that grip strength among adults 60–69 years old in Asia is significantly lower than among their peers in the West (Wang, 2010). The intuitive perception of the decline in physical function in older adults is primarily because of aging rather than frailty. However, upper limb muscle strength and endurance were directly associated with the frailty indicator of hand-grip strength. Therefore, upper limb dysfunction indirectly accelerates the process of becoming frail and affects overall quality of life. This further shows that weak muscle strength in the upper limbs increases the severity of frailty and reduces HRQoL. Similar to the analysis in earlier studies (Chang et al., 2012), frailty status was found in this study to be a prognostic factor of HRQoL. In addition, the need to pay special attention to upper limb function in older adults was highlighted in this study, and the finding that sarcopenia induced frailty is clinically relevant to the HRQoL. The key conclusions of the above discussion are that improving physical activity in the upper limbs and improving frailty status hold the potential to significantly promote HRQoL in older community-dwelling adults.

Recent interventional studies of community seniors have found that grip strength and physical fitness significantly improved after a 12-week physical training program coupled with nutritional measures and family visits (Haider et al., 2017). Another study that focused on women categorized as prefail in the community found that a 1-hour combined exercise training and cooking course designed to increase muscle mass over a 12-week period significantly increased participant grip strength and HRQoL. This emphasizes that upper limb functional impairment poses a significant health problem to older community-dwelling adults (Kwon et al., 2015). Another study found that a 3-month health education intervention using a case manager improved the physical function of older adults who were in the early stages of frailty or considered frail (Chen, 2015). The analysis in this study shows that an exercise training, nutritional intervention, and follow-up management model has the potential to significantly improve muscle strength and physical fitness and thus prevent, delay, or transform the development of frailty and positively affect HRQoL in older community-dwelling adults. Previous studies have shown correlations among lower limb function, frailty status, and HRQoL, which highlights the importance of fall prevention (Chang et al., 2014; Sewo Sampaio et al., 2016). However, this study found that upper limb function greatly affects the ability to perform the fine movements required for self-care. These studies indicate that increasing upper limb function in older adults promotes HRQoL. The findings of this study may further be used as a basis for future research applications in geriatric care. On the basis of our findings, communities should both offer guidance and support to their older residents and promote activities to these individuals that include physical fitness tests. Using organization-appropriate, regular, and effective physical exercises and nutrition-related health promotion courses in community care sites as well as encouraging active participation in community activities will not only enhance daily activity functioning abilities, delay aging, promote interpersonal interactions, and provide social support but also help older adults pay greater attention to their personal physical fitness status and quality of life.

### Conclusions and Limitations

Analysis of the demographic characteristics of the participants indicates that having more chronic diseases, more fall experiences, and a higher BMI have a significant effect on HRQoL. The severity of frailty was positively associated with the severity of impacts on upper and lower extremity muscle strength and endurance, static balance, cardiovascular endurance, upper and lower limb flexibility, dynamic balance and agility, and participation in physical activity. Higher physical activity scores were associated with higher levels of HRQoL, whereas higher frailty scores were associated with lower levels of HRQoL. The prognostic factors of the HRQoL were found to be upper limb strength and muscle endurance and frailty status. Full model testing explained 28% of the variation in HRQoL.

Recommendations for future application include the following:

1. Clinical nursing practice: strengthen the evaluation ability and skills of nursing staff in recognizing the frailty phenotypes using the CHS and the SFT. Nursing staff should have the ability to propose health support measures such as physical exercise and nutritional planning to help identify physical dysfunction and outcomes in older adults with frailty. Furthermore, when necessary, nurses should assist by making referrals to senior care facilities for appropriate treatment and HRQoL improvement measures.
2. Nursing education: train nursing staff on chronic disease management, physical fitness assessment, and the concepts underlying frailty in older adults via standard nursing curricula or continuing education programs. Encourage community nursing personnel to receive relevant training and certification. Trained nursing staff should be able to design intervention activities and curricula for high-risk groups based on assessment results to promote life quality during old age.
3. Policy administration: actively cultivate the ability of community geriatric nursing professionals to provide appropriate guidance to older adults. Develop prevention strategies against aging and physical fitness programs for older adults with frailty and upper limb dysfunction.
4. Academic research: set up intervention research studies on older adults to investigate the empirical effectiveness of compound exercises and nutrition knowledge in improving upper limb function and to identify the related factors. This may further enhance the scholarly understanding of HRQoL at each stage of frailty.

The participants in this study were limited in terms of time and community care site. As the participants were not selected by random sampling, the results are not generalizable to all older community-dwelling adults. As low physical activity and frailty are related to lower participation in community activities, the research data collection may have been affected by sampling bias. To date, a unified standard for indicators of frailty has not been established, and only a few relevant studies concerning the above two parameters have been conducted. In this study, we investigated the correlations between frailty and physical activity, respectively, and HRQoL in older community-dwelling adults. Further investigations should be conducted to identify the intermediary, regulation, and causal relationships among physical activity, frailty status, and HRQoL.
